# ceRNA network construction and identification of hub genes as novel therapeutic targets for age-related cataracts using bioinformatics

**DOI:** 10.7717/peerj.15054

**Published:** 2023-03-23

**Authors:** Yingying Hong, Jiawen Wu, Yang Sun, Shenghai Zhang, Yi Lu, Yinghong Ji

**Affiliations:** Eye Institute and Department of Ophthalmology, Eye & ENT Hospital, Fudan University, Shanghai, China

**Keywords:** Age-related cataract, lncRNA, ceRNA network, Enrichment analysis, Bioinformatics

## Abstract

**Background:**

The aim of this study is to investigate the genetic and epigenetic mechanisms involved in the pathogenesis of age-related cataract (ARC).

**Methods:**

We obtained the transcriptome datafile of th ree ARC samples and three healthy, age-matched samples and used differential expression analyses to identify the differentially expressed genes (DEGs). The differential lncRNA-associated competing endogenous (ceRNA) network, and the protein-protein network (PPI) were constructed using Cytoscape and STRING. Cluster analyses were performed to identify the underlying molecular mechanisms of the hub genes affecting ARC progression. To verify the immune status of the ARC patients, immune-associated analyses were also conducted.

**Results:**

The PPI network identified the FOXO1 gene as the hub gene with the highest score, as calculated by the Maximal Clique Centrality (MCC) algorithm. The ceRNA network identified lncRNAs H19, XIST, TTTY14, and MEG3 and hub genes FOXO1, NOTCH3, CDK6, SPRY2, and CA2 as playing key roles in regulating the pathogenesis of ARC. Additionally, the identified hub genes showed no significant correlation with an immune response but were highly correlated with cell metabolism, including cysteine, methionine, and galactose.

**Discussion:**

The findings of this study may provide clues toward ARC pathogenic mechanisms and may be of significance for future therapeutic research.

## Introduction

As population age around the world trends upward, age-related cataracts (ARC) continue to be the primary cause of reversible severe vision impairment and blindness worldwide, with incidence increasing. The World Health Organization estimated that in 2014, there were 95 million people worldwide who were visually impaired due to cataracts (WHO, 2014), and a 2018 meta-analysis projected that the number of people affected by ARC would increase to 187.26 million (Song et al., 2018). The primary disease mechanisms of ARC formation are metabolic abnormalities and oxidative stress, mainly caused by ageing (Tang et al., 2015). There are also several risk factors for ARC formation including ultraviolet B exposure, some systematic disease factors (diabetes and hypertension), and some lifestyle factors such as smoking, drinking, and malnutrition (Liu et al., 2017). Currently, the only effective treatment method for ARC is the surgical removal of the onset lens and replacement of the intraocular lens. This procedure has a high success rate, but is also expensive. There are currently no effective therapeutic drugs for cataracts. With expensive surgery as the only treatment option available, cataracts are a significant medical and financial burden both at the individual and population levels. Further characterization of the pathogenesis of ARC is essential for developing new therapies.

Non-coding RNA, such as long non-coding RNA, circular RNA, microRNA, and pseudogenes, lack open-reading frames so they cannot be translated into proteins. Long non-coding RNAs (lncRNAs) are RNA molecules that are longer than 200 nucleotides. There are seven main types of lncRNA, including antisense lncRNAs, intronic lncRNAs, bidirectional lncRNAs, intergenic lncRNAs (lincRNAs), enhancer RNAs (eRNAs), and circular RNAs (circRNAs; Beermann et al., 2016). Recent studies have found that lncRNA can interact with proteins, DNA, and RNA, contributing to the pathogenesis of ocular disease (Chen et al., 2022; Zhang et al., 2019). Moreover, lncRNAs have been shown to be differentially expressed in ocular tissue, which may also play a vital role in the pathogenesis of ophthalmic diseases such as corneal disease, glaucoma, cataracts, retinopathy, and ocular tumors (Liu & Qu, 2021; Zhang et al., 2019). The modification of lncRNA is critically involved in cellular senescence and the ageing process, which contribute to the pathological mechanisms of ARC through cell proliferation, apoptosis, migration, and epithelial-mesenchymal transition (EMT; Chen et al., 2022; Jiapaer et al., 2022). For example, previous studies have shown that lncRNA 1-phosphatidylinositol-4,5-bisphosphate phosphodiesterase delta 3-sence RNA 1 (PLCD3-OT1; Xiang et al., 2019a), H19 (Cheng et al., 2019; Liu et al., 2018), myocardial infarction associated transcript (MIAT; Ling et al., 2020; Shen et al., 2016), maternally expressed gene 3 (MEG3; Tu et al., 2020), taurine up-regulated 1 (TUG1; Li et al., 2017a; Shen & Zhou, 2021), glutathione peroxidase 3 (GPX3)-antisense (AS; Tu et al., 2019), NONHSAT143692.2 (Zhou et al., 2020), and antisense non-coding RNA in the INK4 locus (ANRIL; Qi et al., 2019) are all involved in the progression of ARC. The ceRNA hypothesis, introduced by Salmena et al., hypothesizes that lncRNAs could bind to miRNAs competitively, then regulate gene expression at the post-transcriptional level (Salmena et al., 2011). Xiang et al. (2019b) using RNA-sequencing, found that the PLCD3-OT1 lncRNA acts as a ceRNA, preventing ARC by sponging miR-224-5p and regulating PLCD3 expression. However, more research is required to determine the expression patterns and mechanisms of specific ceRNA networks in ARC patients.

In this study, we aimed to identify differentially expressed lncRNAs and the correlated hub genes to construct a differential lncRNA-associated competing endogenous (ceRNA) network and explore the underlying molecular mechanisms and therapeutic targets of ARC patients.

## Methods

### Patient tissue sample collection

ARC patients undergoing uncomplicated cataract surgery by one surgeon (Y.H.J.) at the Department of Ophthalmology of the Eye & ENT hospital (Shanghai, China) in 2014 were included in this study. Patients with other ocular and systemic diseases affecting vision, such as high myopia, uveitis, ocular trauma, retinopathy, diabetes, and hypertension, were excluded. During the capsulotomy procedure, the anterior lens capsules (ALCs) of the ARC patients included in the study were collected in an RNase-free tube and frozen with liquid nitrogen. Three ALCs were mixed in each tube as one sample to reach enough RNA concentration to be able to conduct the study. The control anterior lens capsule specimens, age-matched to ARC patients, were obtained from the Shanghai Red Cross Eye Bank. This study fully complied with the Declaration of Helsinki and received ethics approval from the Fudan University-affiliated Eye & ENT Hospital (Shanghai, China; IRB number: KJ2011-25). Informed consent was obtained from all participants.

### Data collection

At least 500 ng of total RNA was extracted from the sample tissue with Trizol reagent, then the cDNA and biotinylated cRNA were prepared with illumina totalPrep RNA amplification kits (Cat#IL1791). An Illumina BeadChip (HumanHT-12_V4) experiment was conducted to obtain the text data file following the cRNA quality examination. Illumina BeadStudio Gene Expression Module v1.0 normalized the gene expression profiles (GSE213546). The FunRich software (downloaded from http://www.funrich.org) was used in this study to conduct the miRNA-related functions enrichment analysis.

### Differential Expression Genes (DEGs)

The lncRNA expression profiles were retrieved from the transcriptome sequencing data using the Gencode annotation file (https://www.gencodegenes.org/) from Perl software (version 30). The differentially expressed messenger RNA (mRNA) and lncRNA were obtained using the “limma” packages for R (version 4.2; R Core Team, 2022) and R studio (version 2022.02.2; R Core Team, 2022; RStudio Team, 2013) by comparing the normal and ARC groups with an adjusted *p*-value <0.05 after filtering the data with standard —log2 Fold Change—>1. The differently expressed genes were then used to explore the molecular mechanisms of ARC pathogenesis and development and the volcano map and correlation plot of the DEGs were drawn.

### Functional enrichment analysis

In order to annotate specific genes and to identify the biological function and signaling pathways of the DEGs associated with the pathogenesis of ARC, the Gene Ontology (GO) and Kyoto Encyclopedia of Genes and Genomes (KEGG) pathways were analyzed using the “ClusterProfiler” R package (based on *p*-value <0.05).

### Immune infiltration-related analysis

The relative infiltration proportions of 22 immune cells in the ARC patients were estimated using the CIBERSORTx algorithm (https://cibersortx.stanford.edu/). The correlation and influence of these immune cell proportions were then analyzed using the “corrplot” R package and the relative percentage of the immune cells were compared by Wilcoxon test.

The relationship between the expression levels of the hub genes (mentioned below) and the number of immune cells was analyzed using the Spearman method with *p* < 0.05 considered significant.

### The construction of the ceRNA network and PPI network

The potential microRNAs (miRNAs) that interact with the differential lncRNAs were predicted using the Perl software combined with the miRcode file (http://www.mircode.org/, the public transcriptome database). The miRDB (http://www.mirdb.org), mirtarbase (https://mirtarbase.cuhk.edu.cn/ miRTarBase/miRTarBase_2022/php/index.php), and TargetScan (http://www.targetscan.org) databases were used in combination to predict the target mRNA of the miRNA. Next, the intersection of miRNA-predicted-mRNAs and differential-mRNAs (co-identified target genes) was identified and used to construct the ceRNA and protein-protein interaction (PPI) network. Next, the five hub genes with the highest scores were identified using the Maximal Clique Centrality (MCC) algorithm (Chin et al., 2014); a topological analysis method introduced by Chin et al. that is good at predicting the proteins that form the yeast PPI network) from the Cytohubba plugin in Cytoscape (version 3.9.1).

### Gene set enrichment analysis (GSEA) and construction of drug network on hub genes

The expression of each hub gene between the normal and ARC groups were compared and the correlations analyzed. The GSEA of each core gene was also performed to explore the biological signaling pathways (based on *p*-value <0.05). Additionally, protein-drug interaction data were retrieved from the DGIdb database (http://www.dgidb.org) and visualized using Cytoscape to predict potential therapeutic agents for ARC patients.

## Results

### Differentially Expressed Genes (DEGs)

**Figure 1:**
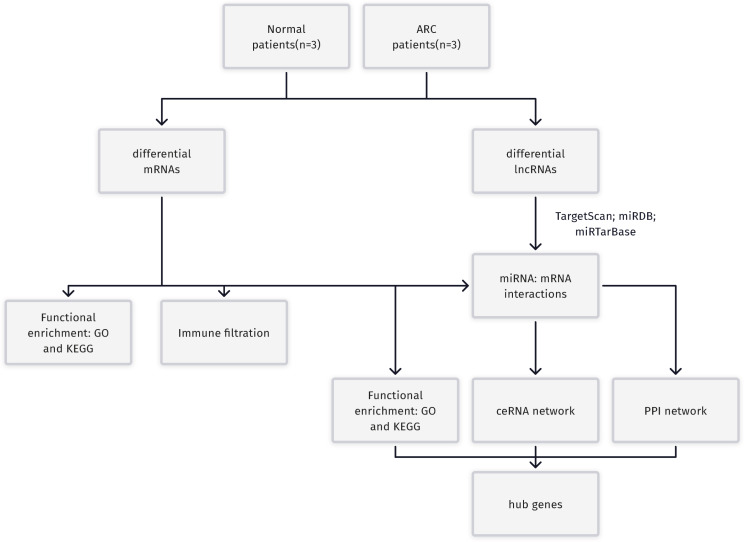
A Flowchart for the identification of potential ARC therapeutic targets. RC, age-related cataract; mRNA, messenger RNA; miRNA, microRNA; lncRNA, long non-coding RNA; GO, Gene Ontology; KEGG, Kyoto Encyclopedia of Genes and Genomes; ceRNA, competing endogenous RNA; PPI, protein-protein interaction.

**Figure 2:**
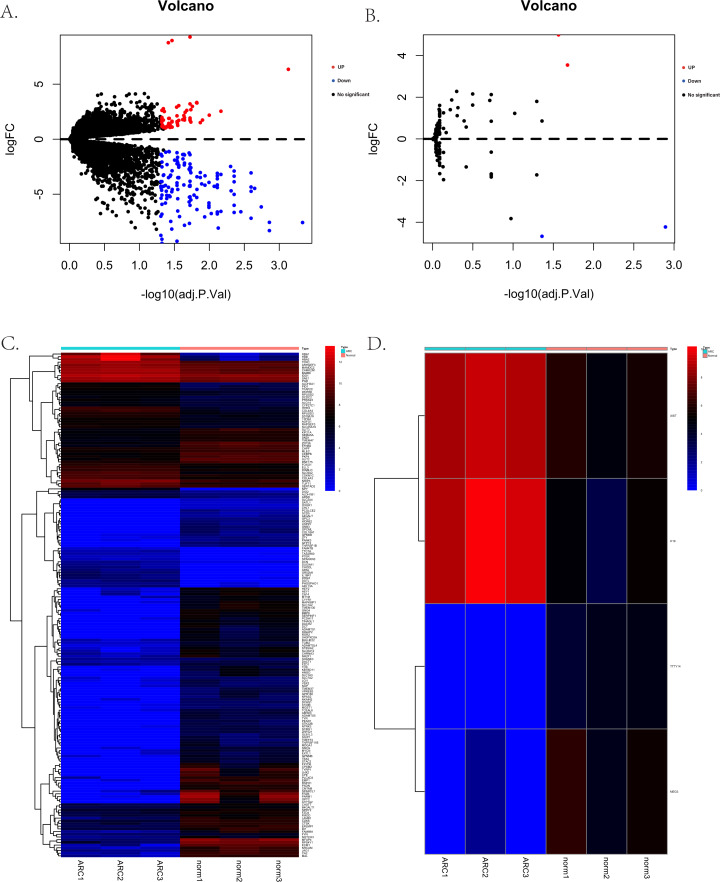
The volcano plots and heatmaps for the differentially expressed genes between the normal group and ARC group (adjusted *p*-value < 0.05 after filtering the data with standard —log2 Fold Change— > 1). (A) The volcano plots of the 187 identified differentially expressed mRNAs, including 56 up-regulated genes (red dots) and 131 down-regulated genes (blue dots); (B) the volcano plots of the four identified differentially expressed lncRNA, including two up-regulated (red dot) and two down-regulated (blue dot) lncRNAs; (C) the heatmap of the differentially expressed mRNAs; (D) the heatmap of the differentially expressed lncRNAs.

The process for identifying potential therapeutic targets for ARC is shown in Fig. 1. After annotating with the Gencode annotation file, 25,023 protein-coding RNAs and 143 lncRNAs were retrieved from the output expression files and used in the differential analysis. With the threshold of adjusted —log2(FC)—>1 and a *p-* value of 0.05, we identified a total of 187 differentially expressed mRNAs, including 56 up-regulated and 131 down-regulated genes. Additionally, four differentially expressed lncRNAs were identified, including two up-regulated—H19 and X-inactive specific transcript (XIST)—and two down-regulated—testis-specific transcript Y-linked 14 (TTTY14) and MEG3. Figures 2A–2D shows the volcano and heatmap plots of the differentially expressed mRNAs (A, B) and lncRNAs (C, D).

### Functional enrichment of DEGs

**Figure 3:**
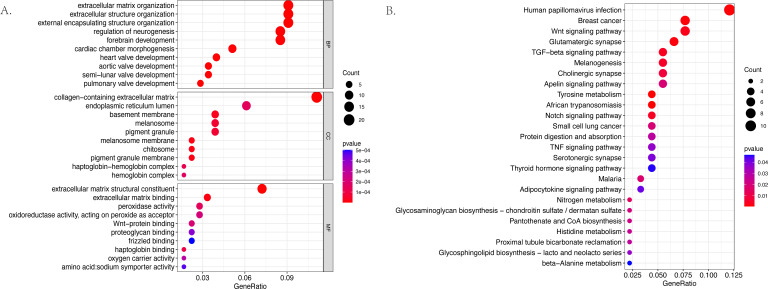
The bubble plot of the enriched functional pathways of the differentially expressed mRNAs between the normal group and ARC group (based on *p*-value < 0.05). (A) The Gene Ontology (GO) analysis shows that the differentially expressed genes are mainly enriched in the biological pathways of extracellular matrix organization and extracellular structure organization; (B) the Kyoto Encyclopedia of Genes and Genomes (KEGG) analysis indicated that the differentially expressed genes are mostly enriched in human papillomavirus infection, breast cancer, and the Wnt signaling pathway.

The GO and KEGG pathway enrichment analyses were conducted on the 187 differentially expressed mRNAs that were identified. The GO analysis identified 559 enriched pathways of significance in the biological processes (BP) category, 55 in the cellular components (CC) category, and 64 in the molecular functions (MF) category. The most significantly enriched terms in each of the categories were then identified (Fig. 3A). The most significantly enriched BP were: extracellular matrix organization, extracellular structure organization, and external encapsulating structure organization. The most significantly enriched CC were the collagen–containing extracellular matrix, endoplasmic reticulum lumen, and basement membrane. The most significantly enriched MF were: the extracellular matrix structural constituent, extracellular matrix binding, and peroxidase activity. The KEGG results identified 25 enriched pathways. The five psathways with the greatest enrichment were: human papillomavirus infection, breast cancer, the Wnt signaling pathway, glutamatergic synapse, and the TGF −beta signaling pathway (Fig. 3B).

### Immune infiltration-related analysis

**Figure 4:**
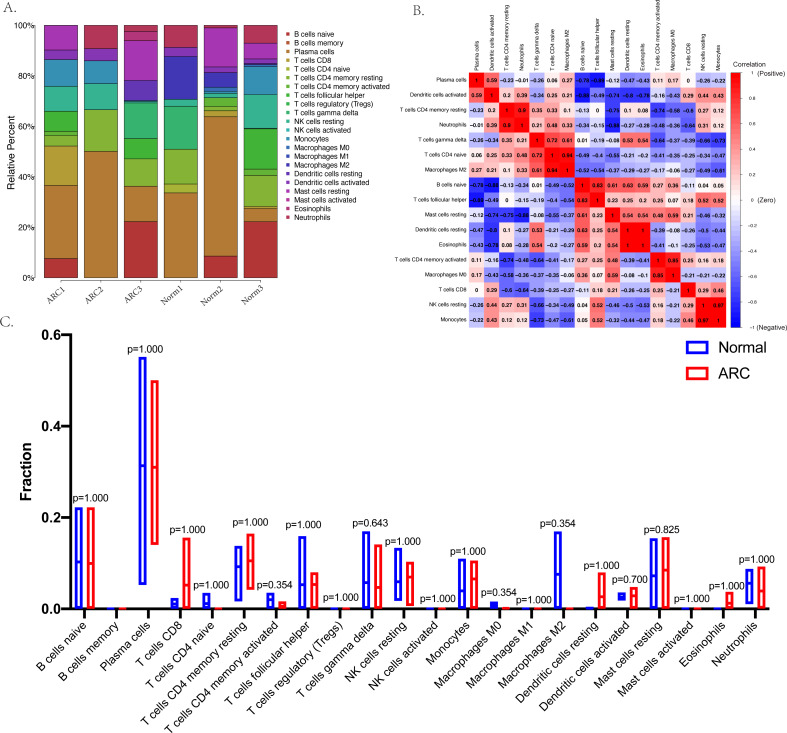
Immune status in the sample analyzed with the CibersortX program. (A) The bar-plot shows the distribution of the 22 types of immune cells in each sample; (B) the correlation plot between the 22 types of immune cells; (C) the box plot comparison (Wilcoxon test) between the control group and ARC group.

The relative infiltration proportions of 22 immune cells in the ARC patients were obtained using the CIBERSORTx algorithm shown in Fig. 4A. The correlation and interaction influence of these immune cell proportions are shown in Fig. 4B. However, the box plot (shown in Fig. 4C) compared the relative number of immune cells between the control group and the ARC group and showed no significant difference.

### The construction of the ceRNA network and PPI network

**Figure 5:**
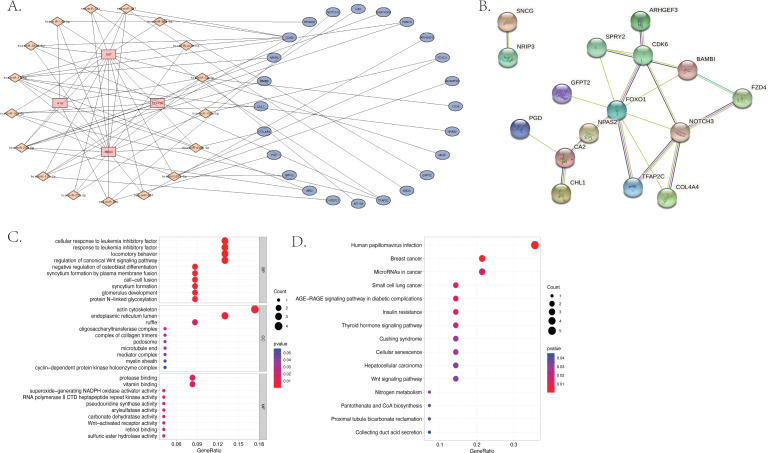
The differentially expressed long non-coding RNA (lncRNA) associated competing endogenous (ceRNA) network, the protein–protein interaction (PPI) network and the functional enrichment of the co-target mRNA. (A) The ceRNA network of 101 pairs of lncRNA-miRNA-mRNA relationships, visualized by Cytoscape; (B) the PPI network of the 24 co-identified target genes, acquired from the intersection of miRNA-predicted-mRNAs and differentially expressed mRNAs, and visualized by the String database (https://cn.string-db.org); (C) the GO analysis and (D) the KEGG analysis of the co-identified target genes.

We used the miRcode database to predict the target miRNA of the four identified differentially expressed lncRNAs. We obtained 202 target miRNAs and 483 mRNA-miRNA pairs. A total of 1,911 target mRNAs and 3,123 miRNA-mRNA pairs were identified by all three databases used (miRDB, mirtarbase, and TargetScan). A total of 24 mRNA were then acquired from the intersection of miRNA-predicted-mRNAs and differentially expressed mRNAs and identified as co-identified target genes. Finally, 101 lncRNA-miRNA-mRNA pairs, including four lncRNAs, 17 miRNAs (hsa-miR-3619-5p, hsa-miR-10a-5p, hsa-miR-17-5p, hsa-miR-24-3p, hsa-miR-20b-5p, hsa-miR-129-5p, hsa-miR-507, hsa-miR-23b-3p, hsa-miR-216b-5p, hsa-miR-107, hsa-miR-135a-5p,hsa-miR-449c-5p, hsa-miR-206, hsa-miR-761, hsa-miR-363-3p, hsa-miR-27a-3p, hsa-miR-140-5p) and 24 mRNA (CDK6, CHL1, ARSJ, TFAP2C, MLEC, ARHGEF3, CA2, NRIP3, COL4A4, CYP27C1, SNCG, NPAS2, FOXO1, PANK3, NOTCH3, PGD, GFPT2, FZD4, SH3PXD2A, RPUSD2, BAMBI, SPRY2, MT1M, ADAMTS5) were obtained and the ceRNA network was visualized using Cytoscape (shown in Fig. 5A). The most enriched BP in the GO analysis was a cellular response to leukemia inhibitory factor, the most enriched CC was actin cytoskeleton, and the most enriched MF was protease binding (Fig. 5C).

**Figure 6:**
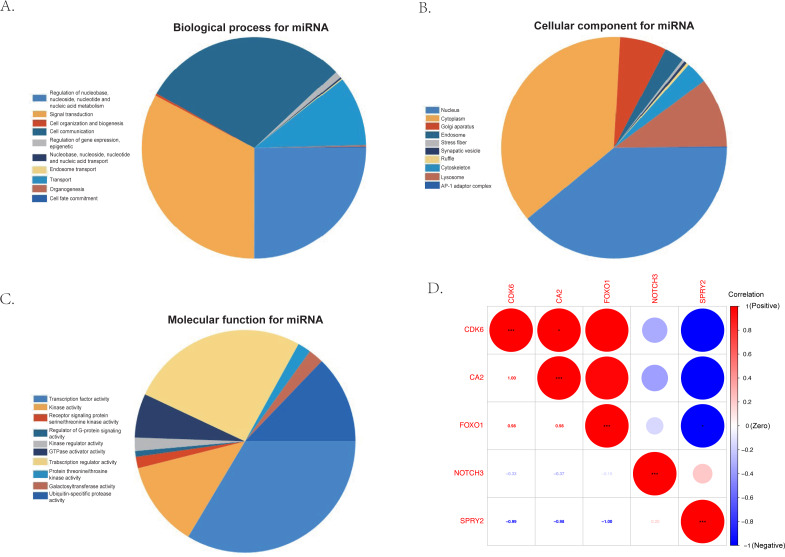
The gene ontology analysis of the predicted miRNA and the correlation of the hub genes. (A) Top 10 biological processes of predicted miRNA. (B) Top 10 cellular components of predicted miRNA. (C) Top 10 molecular functions of predicted miRNA. (D) The correlation plot of the top five hub genes (FOXO1, NOTCH3, CDK6, SPRY2, and CA2) selected by the MCC algorithm of the Cytoscape plugin, Cytohubba.

The KEGG results (Fig. 5D) showed that human papillomavirus infection, breast cancer, and microRNAs in cancer were the top three enriched pathways. The PPI network of the 24 co-identified target genes was generated and visualized using the STRING database and Cytoscape (Fig. 5B). The hub genes, including forkhead box O1 (FOXO1), NOTCH3, Cyclin-dependent kinase (CDK6), sprouty 2 (SPRY2), and Carbonic anhydrase 2 (CA2), were then tagged with the top five MCC scores calculated by the Cytohubba plugin in Cytoscape and selected as potential ARC therapeutic targets. The FunRich software was used to perform a GO enrichment analysis of the nine predicted miRNAs (miR-107, miR-129-5p, miR-135a-5p, miR-206, miR-23b-3p, miR-27a-3p, miR-3619-5p, miR-449c-5p, and miR-761) related to the hub genes. The most enriched BP in the GO analysis was signal transduction (Fig. 6A), the most enriched CC was nucleus (Fig. 6B), and the most enriched MF was transcription factor activity (Fig. 6C). The correlation plot of the expression of the five hub genes is shown in Fig. 6D.

### Gene set enrichment analysis (GSEA)

**Figure 7:**
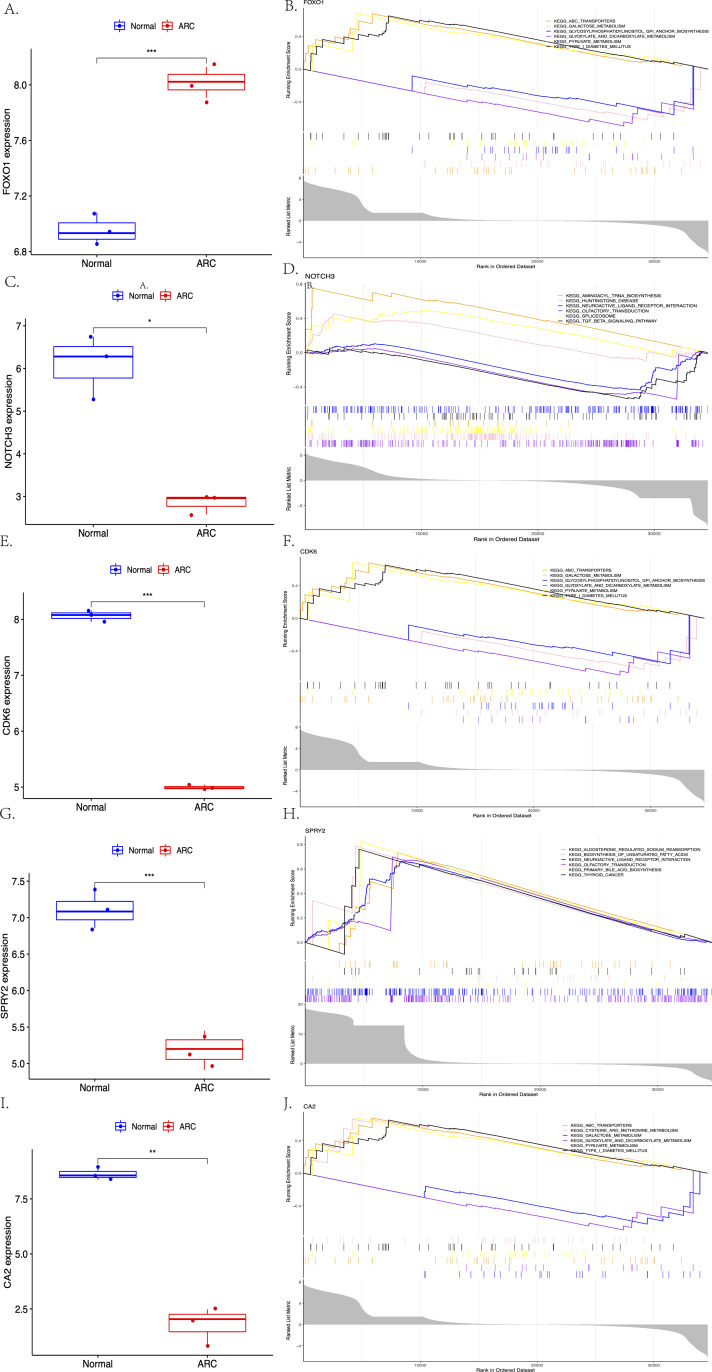
The comparison of the expression of each hub gene between the control group and the ARC group (A, C, E, G, I, *t*-test, *** <0.001 ** <0.01 * <0.05), and the Gene Set Enrichment Analysis (GSEA; based on *p*-value <0.05) of each hub gene (B, D, F, H, J).

The expression of the screened target genes were analyzed between the normal and ARC groups, as shown in Fig. 7 (A, C, E, G, I, ***<0.001, **<0.01, *<0.05). The GSEA of the top six enriched genes—FOXO1 (ABC Transporters, Galactose Metabolism, and Glycosylphosphatidylinositol GPI Anchor Biosynthesis), NOTCH3 (Aminoacyl-tRNA Biosynthesis, Huntingtins Disease, and Neuroactive Ligand Receptor Interaction), CDK6 (ABC Transporters, Galactose Metabolism, and Glycosylphosphatidylinositol GPI Anchor Biosynthesis), SPRY2 (Aldosterone Regulated Sodium Reabsorption, Biosynthesis of Unsaturated Fatty Acid and Neuroactive Ligand Receptor Interaction), and CA2 (ABC Transporters, Cysteine and Methionine Metabolism, and Galactose Metabolism)—was then performed (shown in Figs. 7B, 7D, 7F, 7H, 7J).

### Construction of ceRNA network and drug network on hub genes

**Figure 8:**
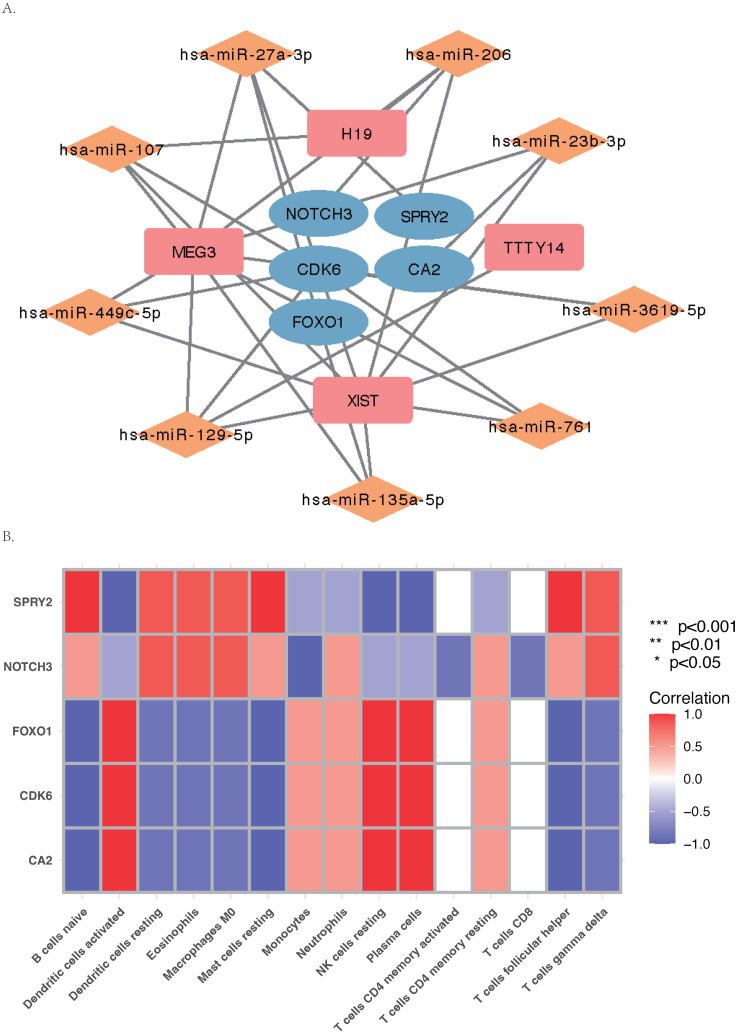
The ceRNA network and Gene Set Enrichment Analysis (GSEA) of the hub genes. (A) The core ceRNA network with four lncRNA, nine mi-RNA, and five hub genes; (B) the correlation between immune cells and the five hub genes.

**Figure 9:**
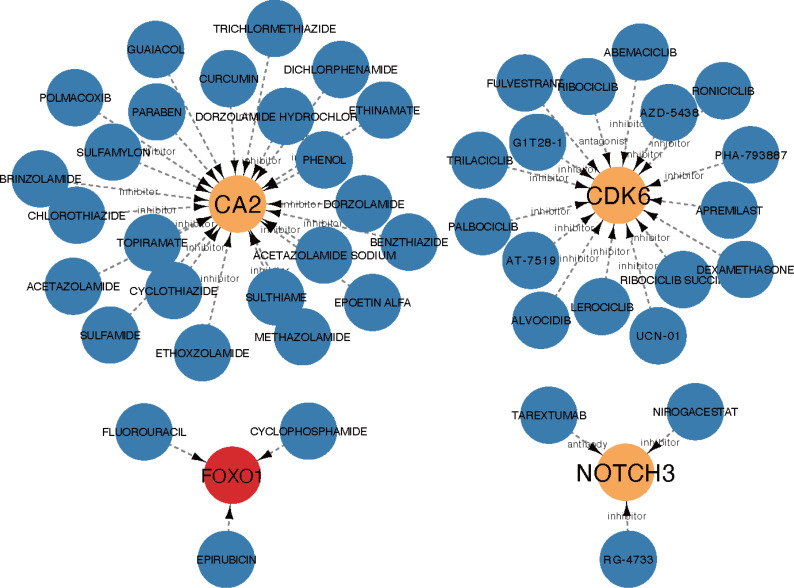
The drug network of the hub genes retrieved by the DGIdb database (https://www.dgidb.org) and visualized by cytoscape; red: up-regulated gene, orange: down-regulated gene, blue: drug.

The core ceRNA network was constructed with five hub genes, the nine miRNAs that interacted with the hub genes, and the four miRNA-targeted lncRNAs (shown in Fig. 8A). Figure 8B shows no significant correlation between immune cells and the five hub genes. A total of 26 drugs that interact with the identified ARC hub genes were acquired from the DGIdb database. Of the top five drugs identified with the greatest interaction score, one (Tarextumab) targets NOTCH3, and four (Dorzolamide, Brinzolamide, Ethinamate, and Sulthiame) target CA2. Four hub genes with drug interactions constitute the drug network visualized by Cytoscape shown in Fig. 9.

## Discussion

In this study, we analyzed the differentially expressed lncRNA in the anterior lens capsules of ARC patients and the control group using a differential expression analysis, functional enrichment analysis, and an immune infiltration-related analysis. We then constructed a ceRNA network and identified five hub genes to serve as possible therapeutic targets for ARC progression.

The GO enrichment of the DEGs in the BP were mainly enriched in extracellular matrix (ECM) organization. Because of the importance of the interactions between the ECM of the lens capsular cells during lens development, the disruption of the ECM or a change in cell signaling, especially transduced by integrins, may stunt lens growth and promote cataracts (Wederell & De Iongh, 2006). The KEGG pathway analysis of the DEGs identified the Wnt and TGF–beta signaling pathways, which is in accordance with the results of previous studies (Chen et al., 2021; Shi & Yang, 2021).

Our analysis showed no statistical differences in the percentage of immune cells between the normal and ARC groups. Our study used an estimated prediction of the abundance of different types of immune cells, and it is important to note that this finding was not experimentally verified and the data was not derived from a single-cell RNAseq experiment. Although a group of resident immune cells of the lens was found to be established during embryonic development and maintained in adulthood, the immune cells surveilling the lens are usually activated during eye injury, lens degeneration, and lens wounding, especially post cataract surgery (Stepp & Menko, 2021).

Competing endogenous RNA (ceRNA) has a complex regulatory network with rich biological functions, which has attracted extensive attention in the academic community. We constructed the ceRNA network using the differentially expressed lncRNA and the intersection of miRNA-predicted-mRNAs and differentially expressed mRNAs. Subsequently, the five hub genes with the highest scores, as calculated by the MCC method, were selected from the co-identified target mRNA of the PPI network. There was no significant correlation between the hub genes and the proportion of immune cells, which was consistent with the results of the immune analysis. Conversely, the GSEA demonstrated that the hub genes were highly correlated with cell metabolism, in line with previous research (Chen et al., 2014; Duncan & Wormstone, 1999; Stambolian, 1988; Truscott, 2005). Finally, the drug network was constructed to show the interaction between the identified proteins and possible drug therapies.

There were four differentially expressed lncRNA identified in this study: H19 and XIST were up-regulated, and MEG3 and TTTY14 were down-regulated in the ARC patients. The H19 lncRNA was also found to be up-regulated in ARC patients in Cheng et al. (2019) and Liu et al. (2018), which is consistent with our study. These studies also found that the protective role of H19 lncRNA was associated with cell proliferation and apoptosis by regulating the miR-29a–TDG axis and miR-675-CRYAA axis. Fibrosis in the lens (EMT process, including the loss of epithelial cell integrity with abnormal proliferation, migration, and the cell morphology changing into more mesodermal-derived mesenchymal-like cells), increasing apoptosis resistance, and exaggerated ECM components production, is featured by the accumulation of excess connective tissue that destroys the normal structure and function of the lens (Lovicu, Shin & McAvoy, 2016).

In an *in vitro* study, Xiong et al. (2022) indicated the novel role of H19 lncRNA in inhibiting TGF- *β*2-induced EMT to prevent lens fibrosis. Previous studies have also demonstrated that Xist lncRNA can play a role in regulating X chromosome inactivation (XCI) and lead to the inheritable silencing of one of the X-chromosomes during female cell development. Xist lncRNA is also involved in tumor development and the progression of other diseases by acting as a ceRNA (Wang et al., 2021). Our study’s functional enrichment results showed no significant enrichment in the X-chromosome, indicating that the ARC pathogenesis may be independent of the X-chromosome. Another study found that Xist lncRNA plays a protective role in diabetic cataracts by promoting cell proliferation and decreasing apoptosis through the miR-34a/SMAD2 (Wang, Zhao & Zhang, 2022). It is unclear in our study whether the Xist up-regulation that was observed was a causative or reactive protective factor in ARC. Another study showed that MEG3 lncRNA is a cataractogenesis molecule through the up-regulation of TP53INP1 in ARC patients (Tu et al., 2020). Though TTTY14 lncRNA has not been found in any cataract pathogenesis, previous studies have shown that it is associated with the progression of cancers (Gong et al., 2020; Kopczyńska et al., 2020; Li et al., 2017b), endometriosis (Bhat et al., 2019), and COVID-19 (Askari, Hadizadeh & Rashidifar, 2022), suggesting the critical role of TTTY14 in biological processes. Therefore, the molecular function of TTTY14 in ARC patients should be further studied.

MicroRNAs (miRNAs) are a class of endogenous short non-coding RNAs (containing ∼22 nucleotides) that are part of the epigenome and post-transcriptional control gene expression through translational repression or mRNA degradation (Cai et al., 2009). The predicted target miRNAs of the hub genes associated the ceRNA network include miR-107, miR-129-5p, miR-135a-5p, miR-206, miR-23b-3p, miR-27a-3p, miR-3619-5p, miR-449c-5p, and miR-761. For example, the upregulated miR-23b-3p , which involved in apoptosis (Liu et al., 2022), autophagy (Zhou et al., 2019), and resistance to oxidative damage (Liang et al., 2020) in ARC *via* translational repression of key molecules, such as homeodomain interacting protein kinase 3 (HIPK3) and silent information regulator 1 (SIRT1). Importantly, the miRNAs were predicted by miRNA related database which need further experiment validation.

FOXO1, one of the members of the FOXO subfamily of Forkhead transcription factors, is known as a cell response regulator to oxidative stress. Up-regulation of FOXO1 has been reported in high glucose-treated human lens epithelium cell (HLEC) lines contributing to cataractogenesis as a cell death-related gene and serving as the target of Lycium barbarum polysaccharide treatment (Yao et al., 2020). Conversely, another study (Zhu et al., 2018) with different glucose concentrations showed that up-regulating FOXO1 can prevent HLECs from oxidative damage induced by high glucose *via* beta-casomorphin-7 treatment. FOXO1 is also associated with choroidal neovascularization, and retina vein occlusions have also been reported (Chen et al., 2019; Zhou et al., 2021b). However, there is a lack of research on the role of up-regulated FOXO1 in ARC pathogenesis.

Notch proteins (NOTCH1-4) are a family of transmembrane receptors that play a vital role in both developmental and cell fate decisions (Artavanis-Tsakonas, Rand & Lake, 1999). The dysregulation of Notch proteins is involved in many diseases, such as cancer, cerebral autosomal dominant arteriopathy with subcortical infarcts and leukoencephalopathy, and pulmonary hypertension (Hosseini-Alghaderi & Baron, 2020). Additionally, Zhou et al. (2021a) proposed that the down-regulation of NOTCH3/Hes1 was related to the apoptosis of lens epithelial cells under cold stimulation, which supports the decreased NOTCH3 expression observed in ARC patients in our study. However, another study suggested that NOTCH3 can be directedly regulated by the toll-like receptor (TLR)-3, contributing to the process of EMT, triggering fibrotic cataracts (Xie et al., 2022). Thus, further studies of the NOTCH3 inactivation mechanism in ARC patients are needed.

It is universally accepted that Sprouty proteins with four mammalian orthologs (SPRY1-4) function as antagonists of receptor tyrosine kinase-induced signal transduction in organisms, which is essential to growth and development (Cabrita & Christofori, 2008). The down-regulation of SPRY2 in patients with ARC has been observed in a number of studies. Previous studies (Lovicu, Shin & McAvoy, 2016; Shin et al., 2012) have shown that SPRY2 is a protective factor as a negative regulator of transforming growth factor *β*-induced EMT and cataract formation, likely by regulating ERK1/2 and Smad2 (Tan et al., 2016; Zhao et al., 2022). Studies have also shown that SPRY2 interacts with microRNA (Liu et al., 2019; Liu et al., 2021), suggesting that it may play a crucial role in the EMT of lens epithelial cells in ARC patients.

The down-regulation of CDK6 and CA2 were also identified in our study. CDK6, which is highly correlated with the cell cycle, constitutes a complex with cyclin D and CDK inhibitor to control the G1 checkpoint through the phosphorylation of the retinoblastoma protein (pRb; Lukas, Bartkova & Bartek, 1996). A study exploring the expression and activity of CDK cells during lens differentiation (developing rat lenses) showed that Cdk6 was not expressed in lens fiber cells or epithelial cells during lens differentiation (Gao et al., 1999), but there has been little study on the relationship between CDK6 and ARC.

There are 15 known human isoforms of carbonic anhydrase (CA) with different functions and distributions, which belong to a class of metalloenzymes that catalyze carbon dioxide into bicarbonate. Human CA variants have been linked to glaucoma, macular edema, ulcers, obesity, and cancer (Cabaleiro-Lago & Lundqvist, 2020). A study (Wistrand, 1999) investigating human lens CA demonstrated that the CA activity originates from CA 1, 2 and 3 in the cytoplasm , and from CA4 in the plasma membranes of lens epithelium and fibers in normal patients, and there was no CA activity observed in the supernatant of senile cataract lenses. As studies (Wistrand, 1999; Wistrand, 2000) have shown that the chronic intake of CA inhibitor does not seem to induce lens opacification (signs of cataract), we hypothesize that the down-regulation of CA2 observed in our study may be a protective response to oxidative stress.

Some limitations in this study should be recognized. First, our results require further validation through cell and animal experiments. Second, the samples are limited and lack clinical signatures (such as postoperative vision, surgery complication, and visual quality), which needs further study by enlarging the samples combined with clinical information.

## Conclusion

The differentially expressed lncRNAs and hub genes identified in this study have the potential to serve as therapeutic targets for ARC patients based on cell metabolism.

### RNA-seq power calculation

Not applicable. This is study used an Illumina BeadChip microarray of transcriptome sequencing with three biological samples as minimum replicates to conduct comparative gene expression analyses (Conesa et al., 2016).

##  Supplemental Information

10.7717/peerj.15054/supp-1 Supplemental Information 1 Miame checklist

10.7717/peerj.15054/supp-2 Supplemental Information 2 Raw data

## Additional Information and Declarations

### Microarray Data Deposition

The following information was supplied regarding the deposition of microarray data: The data is available at GEO: GSE213546.

### Data Availability

The following information was supplied regarding data availability: The raw data are available in the Supplementary File.
