# Supplementary material for: ceRNA network construction and identification of hub genes as novel therapeutic targets for age-related cataracts using bioinformatics
Source: PeerJ. 2023 Mar 23;11:e15054. doi: 10.7717/peerj.15054 (PMC10040182; doi:10.7717/peerj.15054)
Supplement: Supplemental Information 1 [file peerj-11-15054-s001.docx]

**Miame Checklist**

**Part 1 Experiment description**

1. Homo sapiens
2. Normal human vs age-related cataract patients
3. three ARC samples and three age-matched normal samples
4. the anterior lens capsules of the included ARC patients and normal lens
5. The inclusion and exclusion criteria were ARC patients undergoing uncomplicated cataract surgery. The control anterior lens capsule specimens with age-matched to ARC patients was obtained from the Shanghai Red Cross Eye Bank.

**Part 2 Array design.**

1. HumanHT-12_V4 as the platform
2. BIOTIN——The average biotin control intensity across all probes; CY3_HYB——The average CY3_HYB control intensity across all probes; HIGH_STRINGENCY_HYB——The average HIGH_STRINGENCY_HYB control intensity across all probes; HOUSEKEEPING——The average HOUSEKEEPING control intensity across all probes; LABELING——The average LABELING control intensity across all probes; LOW_STRINGENCY_HYB——The average LOW_STRINGENCY_HYB control intensity across all probes; NEGATIVE——The average NEGATIVE (background)control intensity across all probes

**Part 3 Samples**

 RNA was extracted with Trizol reagent, followed by clean-up and DNase I treatment with Illumina TotalPrep RAN amplicaiton kit in accordance with the prescribed protocol provided with the kit.

Biotinylated cRNA were prepared with the Illumina TotalPrep RAN amplicaiton kit for Illumina arrays

**Part 4 Hybridizations**

1. hybridize
   1. Sentrix BeadChip : Hyb chamber 58℃;
   2. GEX-HCE
2. Room-temperature Incubation
   1. Wash E1BC buffer
   2. 250 ml stain dish
3. High-temperature wash
   1. High-temp wash buffer
   2. RNase-free water
   3. Hybex^TM^ Heating Base
   4. Hybex^TM^ Waterbath
4. First room-temperature wash
   1. Wash E1BC buffer
   2. 250ml stain dish
5. Ethanol Wash
   1. 100% Ethanol
   2. 250ml stain dish
6. Second room-temperature wash
   1. Wash E1BC buffer
   2. 250ml stain dish
7. Block
   1. BeadChip Wash Tray
   2. Block E1 buffer
8. Detect Signal
   1. BeadChip Wash Tray
   2. SA-Cy3 FluoroLink ^TM^Cy3^TM^
   3. Block E1 buffer
9. Third room-temperature wash
   1. Wash E1BC buffer
   2. 250ml stain dish
10. Dry

**Part 5 Measurements**

1. Read and Extract Image
   1. Illumina BeadChip Reader
2. Analyze Data
   1. Illumina BeadStudio Application
3. Produce Output

**Part 6 Normalization controls**

The data were normalised using quantile normalisation with GenomeStudio Gene Expression Module v1.0

Normalization algorithms transform sample signals in order to minimize the effects of variation arising from non-biological factors. The BeadStudio Gene Expression Module offers four routines that are described in the following sections.

For all algorithms, normalization is computed with respect to a mathematically calculated “virtual” sample that represents averaged probe intensities across a group of samples.

In the cases of spline and rank invariant normalizations, the virtual sample is computed based on the content of the reference group. If there is no reference group, the first group in the list of groups displayed in the **Project Group** pane is used for group analysis.

For BeadChip analysis, the virtual sample is computed based on the content of the first alphanumeric entry in the upper-left area of the Matrix pane. For the hyb controls and average methods, all samples in the experiment are averaged to produce the virtual sample.
